# Influence of Alloying Elements on the Dynamic Recrystallization of 4 wt.–% Medium Manganese Steels

**DOI:** 10.3390/ma13225178

**Published:** 2020-11-17

**Authors:** Alexander Gramlich, Hanne Schäfers, Ulrich Krupp

**Affiliations:** Institut für Eisenhüttenkunde of RWTH, Aachen University, 52072 Aachen, Germany; hanne.schaefers@iehk.rwth-aachen.de (H.S.); krupp@iehk.rwth-aachen.de (U.K.)

**Keywords:** forging steels, medium manganese steels, hot deformation, flow curves

## Abstract

The hot deformation behaviour of air-hardening martensitic forging steels (of type 1.5132) is presented. The newly developed steels are characterized through dilatometric tests as well as through microstructure analyses with LOM and SEM and hardness measurements. Additionally, the influence of alloying elements on the flow curves at high temperatures is discussed. It is demonstrated that the higher alloying content does not increase the equivalent stresses in comparison to the reference alloys and contrariwise reduces the offset for dynamic recrystallization at temperatures below 1100 ${}^{\circ }C$. Furthermore, the effect of different alloying elements on the strain hardening behaviour during hot compression of 4wt.–% medium manganese steels is presented. It is shown that boron reduces the offset for dynamic recrystallization if present in solid solution, while the combined addition of titanium and niobium annihilates the solid drag effect on the prior austenite grain boundaries.

## 1. Introduction

Martensitic steels are broadly used in the forging industry especially if the applications require a decent balance of strength and toughness. Most martensitic steels are produced through a three step heat treatment, consisting of austenization, quenching to room temperature in water or oil followed by tempering. This heat treatment is necessary as classic quench and tempering steels (Q + T), like 42CrMo4 (or AISI 4140) need high cooling rates after the austenization, in order to prevent large carbide formation. As the Q + T heat treatment is very energy intensive, a shortening of this process is desirable. These circumstances were the drive for a wide range of steel development during the last decade [1], starting with precipitation hardening ferritic pearlitic (PHFP) and continuing with bainitic forging steels [2]. PHFP steels are cooled directly from the forging heat and need no further heat treatment. However, the mechanical properties are inferior compared to Q + T steels. Bainitic forging steels, especially transformation induced plasticity (TRIP) assisted alloys [3], achieve properties very close to those of Q + T steels, but their cooling from the forging heat is not easy and specific cooling routes have to be applied. This complicates the process control immense, specifically for forging parts with large diameters. Recently, air-hardening ductile forging steels (AHDs) were added to the list, which achieve their martensitic microstructure through air-cooling from the forging heat, but show similar static mechanical properties as Q + T steels [4]. These materials belong to the group of medium manganese steels (MMnS), as the martensitic microstructure is achieved by an addition of manganese of 4 wt.–%. Additionally, these alloys show a cyclic material behaviour [5] being superior to standard Q + T steels disclosing new potentials in modern automotive lightweight design. Most application and research on MMnS is focussed on sheet application, which is why the hot deformability properties of these alloys are generally not discussed. Through the broadening of the application to the field of forging, the interest in these properties increases as the addition of a large amount of manganese complicates the forging process, because manganese increases the high temperature strength and decreases the formability. First results on this issue [6] show that an increasing carbon concentration (from 0.1wt.–%–0.3wt.–%) decreases the equivalent stress. Conversely, an addition of manganese increases the equivalent stress (investigated range: 3wt.–%–10wt.–%), due to the interaction between substitutional dissolved manganese and lattice vacancies leading to increased activation energy for plastic deformation [7]. Stieben et al. [6] showed, that an increase of carbon in the investigated range decreases the minimum temperature, where dynamic recrystallization occurs from 1000 ${}^{\circ }C$–800 ${}^{\circ }C$. Besides the equivalent stress, the ability of the material to exhibit dynamic recrystallization is important as well as the recrystallization leads to dislocation annihilation and new grain formation [8]. Several studies have been published in the past, investigating the influence of alloying elements like molybdenum [9,10], aluminium [11], titanium [9,12,13] and niobium [9,12,14,15] on the microstructural features during hot deformation, especially dynamic recrystallization (DRX), but none of these studies investigated materials with higher manganese concentrations being of interest for the present applications. The deformation behaviour of MMnS is only characterized for sheet products [16,17,18] using tensile test. The aim of this study is the investigation of the flow behaviour of this alloy group at elevated temperatures, with a special focus on the influence of the alloying elements on the flow curve and resulting microstructural features.

## 2. Materials and Methods

**Figure 1:**
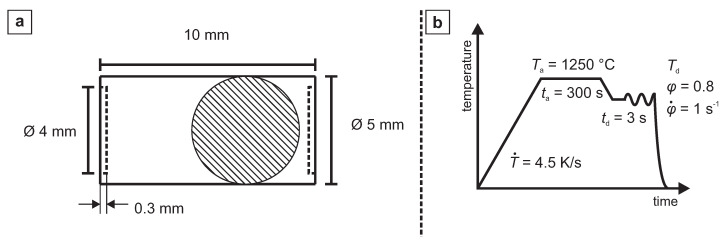
Information on dilatometry testing: (**a**) geometry of cylindrical dilatometry samples with lubrication pockets. (**b**) Time-temperature plot of the dilatometry experiments, with heating rate $\dot{T}$, austenization temperature $T_{a}$, austenization time $t_{a}$, deformation temperature $T_{d}$, holding time before deformation $t_{d}$, deformation degree (equivalent strain) φ, and strain rate $\dot{\varphi }$.

Five alloys were designed using results from previous studies [6] and MatCalc computational thermodynamics software (version 6, MatCalc Engineering GmbH, Vienna, Austria). The base concept (0.15 wt.–%– 0.19 wt.–% carbon, 4 wt.–% manganese, 0.5 wt.–% silicon and 0.035 wt.–%) was originally modified by different amounts of molybdenum, titanium, boron and aluminium in order to investigated the influence of these elements on grain boundary embrittlement [4]. The chemical composition of the investigated alloys is displayed in Table 1. Casting trials (80 kg) were done in a vacuum induction furnace. Afterwards, the ingots were homogenized and the cross sections were reduced significantly (from 19,600 mm^2^ to 3600 mm^2^) by means of hot forging at 1200 ${}^{\circ }C$. The deformation behaviour was investigated with compression test using a deformation dilatometer type Bähr ‘DIL-805A/D’ (TA Instruments, New Castle, DE, USA). Material for cylindrical dilatometer samples (Figure 1) with lubrication pockets was extracted from the transition region between surface and center of the forged rods. For microstructure and hardness investigations all the samples were embedded in epoxy resin and mechanical ground with SiC paper up to 4000 grid. Subsequently, all samples were polished with 3 μm and 1 μm diamond suspension. Dependent on the following investigations, the samples were etched with picric acid for visualizing the prior austenite grain boundaries (pγ-GB) or with nital acid for revealing the microstructure. The prior austenite grain size was characterized by linear intercept method. For each sample, at least 100 grains were included in the measurement. Scanning electron microscopy (SEM) was done on a Zeiss Sigma FE-SEM (Carl Zeiss AG, Oberkochen, Germany) using acceleration voltages from 5kV to 20 kV. Additional data [6] from an industrially manufactured reference steel (ref) of grade 42CrMo4 (AISI 4140) is added for comparison.

## 3. Results

### 3.1. Microstructure

**Figure 2:**
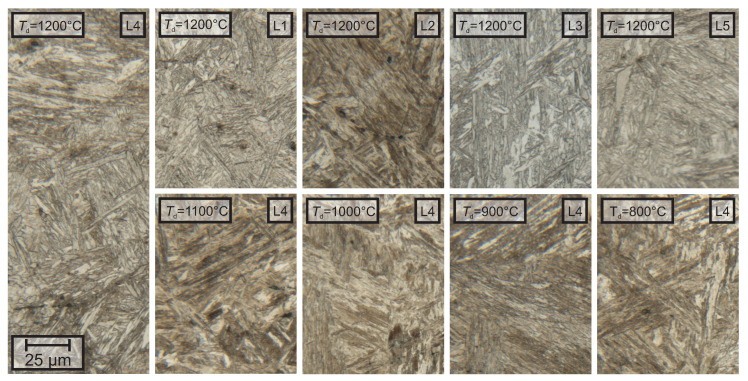
The micrographs show exemplary the microstructure for the investigated samples revealed by nital etching. No significant differences were found in the microstructure if the different alloys were compared with another nor when then different deformation temperatures were compared.

Investigations with light optical microscopy (LOM) show that all samples had a martensitic matrix with regions of increased carbide density. Only minor differences could be found between the different materials or the different forging temperatures. The lath morphology of the martensite was more pronounced at higher temperatures for lab-1. With increasing temperature, the single packets got more distinguishable from each other and the size of the laths increased slightly. This transition mostly took place between 900 ${}^{\circ }C$ and 1100 ${}^{\circ }C$. The laths of lab-2 were less pronounced and with increasing temperature more round and blocky laths were observable. While the evolution of lab-3 could be described similarly to lab-1, lab-4 showed a less homogeneous microstructure than the alloys described so far. Especially at 1100 ${}^{\circ }C$, large blocky regions were observable. Lab-5 shows very fine martensite laths with a continuous increase of the size of the laths from 800 ${}^{\circ }C$ to 1200 ${}^{\circ }C$. The microstructure of all investigated materials after deformation at 1200 ${}^{\circ }C$ and the microstructure evolution of alloy lab-4 are shown in Figure 2.

**Figure 3:**
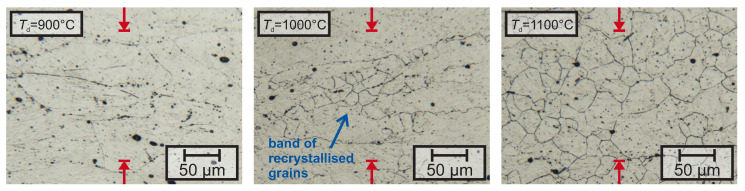
The micrographs show the prior austenite grain boundaries of lab-4 after deformation at 900 ${}^{\circ }C$, 1000 ${}^{\circ }C$ and 1100 ${}^{\circ }C$, revealed by picric etching. The direction of deformation is indicated by the red arrows.

The investigations of the prior pγ-GB reveal fully recrystallized grains after deformation at 1100 ${}^{\circ }C$ and 1200 ${}^{\circ }C$ for lab-1 to lab-5. Deformation at 800 ${}^{\circ }C$ and 900 ${}^{\circ }C$ lead to suppression of recrystallization, as the austenite grains were heavily elongated. After deformation at 1000 ${}^{\circ }C$, only lab-1 to lab-3 show fully recrystallized grains, while alloys lab-4 and lab-5 were showing only bands of recrystallized grains, besides the deformed grains as shown in Figure 3.

While the prior austenite grain size (pγ-GS) of lab-1 showed an increase with increasing deformation temperature (from 31 μm at 800 ${}^{\circ }C$ until 56 μm at 1200 ${}^{\circ }C$) lab-2 showed a consistent pγ-GS with a maximum of 45 μm at 1100 ${}^{\circ }C$ and a minimum of 39 μm at 800 ${}^{\circ }C$ and 1200 ${}^{\circ }C$. Contrary to the small differences for lab-1 and lab-2, lab-3 to lab-5 showed a much higher grain sizes for deformation at 800 ${}^{\circ }C$ and 900 ${}^{\circ }C$ compared the higher temperatures. With increasing temperature, the size decreased up to a temperature of 1100 ${}^{\circ }C$, while a further increase to 1200 ${}^{\circ }C$ led to an increase of the pγ-GS for these alloys. All measured pγ-GS data are shown in Table 2.

**Figure 4:**
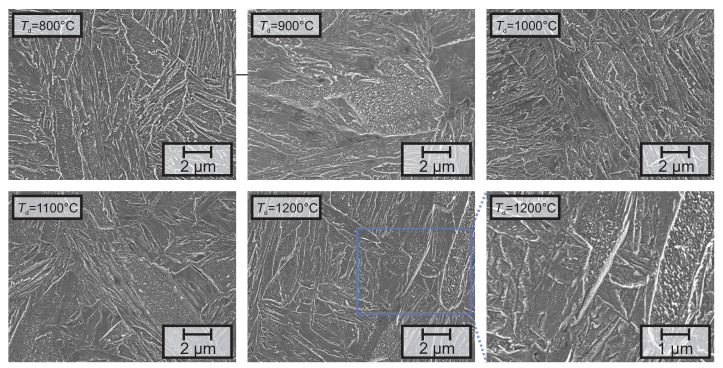
SEM-micrographs of lab-5, revealing the martensitic microstructure after different deformation temperatures (*T*_d_). Besides the martensitic laths, every sample shows regions with a high density of small precipitates, presumably carbides.

SEM-investigations Figure 4 revealed that besides the small differences already noted by LOM, no additional changes in microstructure were introduced by different deformation temperatures. Besides the martensitic matrix, all samples contain regions with increased density of small precipitates. Size, morphology and number density of these precipitates indicate that these particles are carbides.

### 3.2. Deformation Behaviour

**Figure 5:**
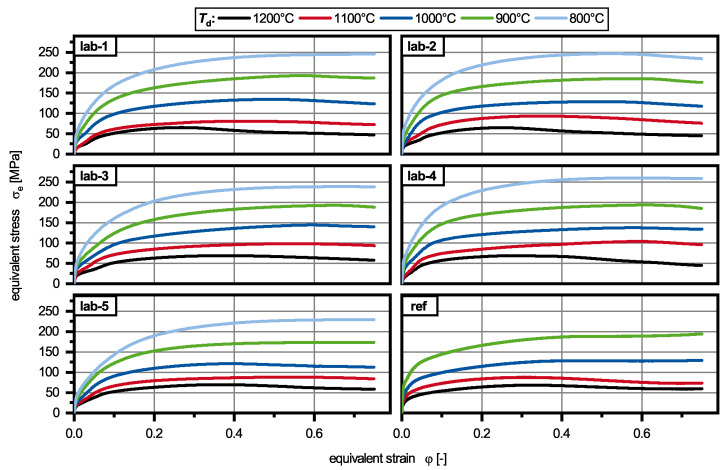
Influence of the deformation temperature on the flow curves of alloys lab-1 to lab-5 and the reference alloy (ref).

The deformation of the laboratory melts revealed that the different alloying concepts showed comparable equivalent stress levels which are comparable to the reference alloy 42CrMo4 (Figure 5). As expected, the equivalent stress increased with decreasing deformation temperature *T*_d_. In the beginning of the deformation all flow curves were characterized by a strong work hardening until the work hardening got weakened by dynamic recovery (DRV). For most experiments DRX was observed leading to a decrease of the equivalent stress at high deformations.

**Figure 6:**
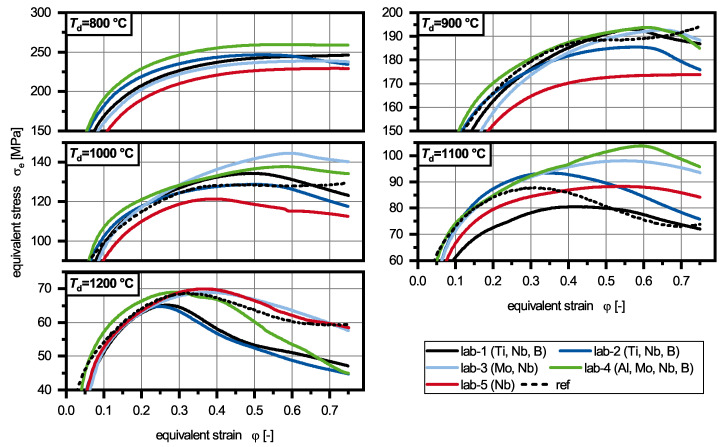
Influence of the chemical composition on the flow curves of alloys lab-1 to lab-5, determined at deformation temperatures from 800 ${}^{\circ }C$–1200 ${}^{\circ }C$.

If the alloying concepts were compared directly with each other, several features could be observed in correlation with the deformation temperature *T*_d_ (Figure 6). The lowest deformation temperature (800 ${}^{\circ }C$) resulted in the highest maximum equivalent stresses between 250 MPa and 360 MPa for alloys lab-5 and lab-4, respectively. Only lab-2 showed stress peaks which indicates DRX, while all other alloys only showed signs of DRV. An increase of *T*_d_ to 900 ${}^{\circ }C$ led to a more pronounced peak for lab-2 and small peaks for lab-1, lab-3 and lab-4. Only lab-5 still showed DRV. At 1000 ${}^{\circ }C$ all materials showed DRX, while the molybdenum-containing alloys (lab-3, lab-4) exhibited the highest peak stresses and peak strains. This was also observed for *T*_d_ = 1100 ${}^{\circ }C$. After deformation at 1200 ${}^{\circ }C$ the materials which were alloyed with titanium show different flow curves than the other ones, with smaller peak stresses and peak strains. The aluminium-containing materials (lab-4) showed a similar flow curve to lab-3 and lab-5, but due to higher strain hardening during the first stages of deformation the peak strain was smaller than these alloys. In general, lab-4 had the highest strain hardening rate at any deformation temperatures.

In comparison to the reference alloy, it can be said that no increase of equivalent stress could be observed for the other alloys in the early stages of deformation. This was in contrast to the expected solid solution hardening due to manganese. The maximum equivalent stresses are displayed in Table 3. For low temperatures (900 ${}^{\circ }C$ and 1000 ${}^{\circ }C$), DRX was observed for lab-1 to lab-5 while ref showed an increase of equivalent stress again at higher deformation temperatures. At 1200 ${}^{\circ }C$, no large difference could be found between ref and lab-3 to lab-5. Only at 1100 ${}^{\circ }C$ ref showed a very small peak strain combined with a small peak stress indicating an early start of DRX.

### 3.3. Hardness

**Figure 7:**
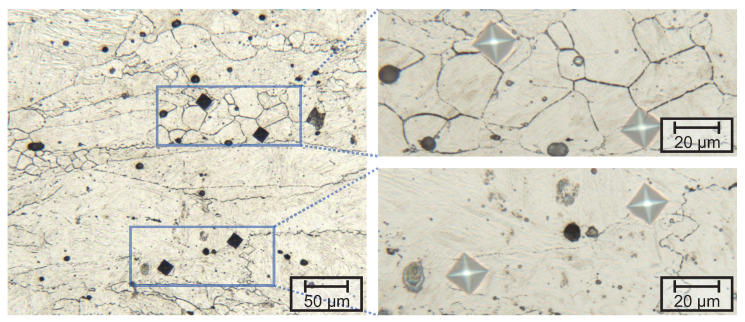
Micrographs of micro hardness indentations (HV0.1) in alloy lab-4 deformed at 1000 ${}^{\circ }C$.

Vickers hardness measurements were performed on the samples after the dilatometric tests in order to estimate the influence of the forging temperature on the mechanical properties. The obtained hardness values are summarized in Table 4. The hardness of all investigated laboratory melts decreased with increasing deformation temperature. Some samples showed small deviations from this trend (lab-5, forged at 1100 ${}^{\circ }C$), but these deviations were within the measuring inaccuracy. The highest hardness was measured for lab-1 at every deformation temperature, followed by lab-3 and lab-5, and finally lab-2 and lab-4. Additional microhardness measurements were performed in order to investigate hardness gradients in only partially recrystallized samples. The micro hardness measurements were performed on the sample of alloy lab-4 which was deformed at 1000 ${}^{\circ }C$. As demonstrated in Figure 7 no significant hardness gradient was found; all indentations revealed a hardness of approximately 460 HV0.1.

## 4. Discussion

The microstructures obtained for medium manganese steels after air-cooling are complex and several features have to be considered to differentiate between martensite or bainite. However, higher magnifications show that the observed carbides are oriented randomly, which is an indication of self-tempered martensite [19]. Additionally, the measured hardness can be correlated with the CCT diagrams of the investigated alloys [20] proving that the present microstructure is indeed martensite and not lower bainite. The gain in hardness with decreasing temperature cannot be explained by the change of pγ-GS size of the materials nor by the residual stored dislocation density from the deformation. As demonstrated by the microhardness indentations (Figure 7) identical hardness was observed in recrystallized and deformed grains. This demonstrates that the dominant factors contributing to the strength is the solution hardening of the martensitic matrix through carbon. Smaller deformation temperatures are leading to reduced cooling times and therefore less carbon diffusion and precipitation. The large pγ-GS of lab-4 after deformation at temperatures below 1000 ${}^{\circ }C$ might be explained by the high aluminium concentration and resulting interactions with other precipitates. As aluminium is a nitride-forming element and alloyed in a much higher concentration than for example niobium, aluminium might trap all nitrogen leaving the matrix technically nitrogen free. As the solution temperature of niobium carbonitrides depend on the amount of nitrogen (with highest solution temperature for NbN), it can be assumed that no niobium precipitates were present during the austenization, which caused extensive grain growth. The missing niobium precipitates also explains the comparable low peak strain ε_p_ in lab-4, as dislocation pinning through particles is known to retard DRX [21]. Additionally, the high concentration of aluminium in lab-4 (0.5 wt.–%) explains the higher strain hardening rate. The material contains fine homogeneously distributed aluminium nitrides [4], which are known to decrease the deformability of steels [11]. The materials deformed at 800 ${}^{\circ }C$ (Figure 6) indicated that boron accelerates the start of DRX, as only lab-2 shows a significant peak at this temperature. Lab-1 which is also alloyed with boron does not show this effect, which might be caused through the reduced concentration (16 ppm) compared to lab-2 (57 ppm). As the titanium concentration is to low to protect all boron from nitrogen, the amount of solute boron is not sufficient in lab-1. These interactions were also observed in microalloyed steels with conventional manganese concentrations [13]. Lab-3 showed higher equivalent stresses in the temperature range from 800 ${}^{\circ }C$ to 1100 ${}^{\circ }C$ in comparison to lab-5. As these alloys can only be differentiated by the molybdenum concentration (lab-3: 0.2 wt.–%), the solute hardening through molybdenum is observed for the investigated materials. Additional, the peak strain was increased at deformation temperatures from 1000 ${}^{\circ }C$ to 1200 ${}^{\circ }C$ showing that molybdenum effectively retards DRX, as reported before [10]. The offset reduction of the DRX through titanium is caused by the size and the composition of the complex (Ti,Nb)(C,N) precipitates. The addition of titanium normally leads to a retardation of DRX [9], through the pinning of pγ-GS effect through Ti(C,N) particles [22]. As previously reported by Ma et al. [12], combined additions of niobium and titanium are as well expected to effectively retard DRX through pinning of pγ-GS by titanium and niobium precipitates. However, in the investigated materials the majority of titanium nitrides have diameters on the μm-scale for which no effective grain boundary pinning can be expected, leaving the positive effect on the DRX questioned. Additionally, titanium nitrides act as nucleation sites for niobium carbides [4,15], annihilating the effect for this type of precipitates as well. Therefore, only the titanium-free alloys (lab-3 to lab-5) have precipitates, which may influence the DRX. The dissolution of niobium precipitates during the heat treatment cannot explain the observed curves, as solute niobium was reported to be the element which increases strain hardening the most [21,22] and precipitates were reported to retard the DRX more effectively, than the solute drag effect [14]. It has to be noted that the absolute values might be influenced by statistic fluctuations, especially at higher deformation temperatures, as only one experiment for each parameter set was carried out. However, the qualitative effects correspond well with the literature. In comparison to the reference alloys, it can be said that no general hardening through manganese was observed for the investigated materials. As reported above, only in the rage of 1100 ${}^{\circ }C$ a higher deformability for ref can be expected due to the early start of DRX.

## 5. Conclusions

The deformability of alloys is influenced by the forging parameters and the interactions of the alloying elements with dislocations, causing features like dynamic recovery or dynamic recrystallization. Therefore, it is of great importance to understand the different interactions between forging parameters and microstructural features in newly developed materials. From the present study the following conclusions can be drawn:

- Contrary to the expectations, no decisive increase of strain hardening was observed in the steels containing 4 wt.–% manganese in comparison to the reference alloy 42CrMo4. Only small differences, dependent on the temperature, were found, caused by different DRX mechanisms.
- No retardation of DRX was observed for titanium- and niobium-containing MMnS. This is caused by the size of the titanium nitrides (approximately 10 μm), which prevents the titanium nitrides from retarding DRX by itself. Additionally, the titanium nitrides act as nucleation sites for niobium carbides which annihilates their interaction with DRX as well.
- If present in solute solution, boron decreases the offset for dynamic recrystallization.
- The prior austenite grain size (from 30 μm to 140 μm) has no measurable influence on the micro hardness of the investigated materials. However, the influence on the Charpy impact energy or tensile test properties have not been investigated and should be addressed in future investigations.

## Figures and Tables

**Table 1:** Chemical composition of the laboratory (lab-1 to lab-5) melts and the reference alloy 42CrMo4 (ref). All concentrations are given in wt.–%.

Alloy	C *	Si	Mn	P	S *	Al	Cr	Mo	Ti	Nb	B
lab-1	0.19	0 50	4.02	0.008	0.011	0.031	0.11	0.02	0.020	0.035	0.0016
lab-2	0.17	0.50	3.99	0.010	0.009	0.025	0.11	0.02	0.020	0.033	0.0057
lab-3	0.15	0.49	4.02	0.011	0.009	0.027	0.12	0.20	<0.003	0.035	<0.0005
lab-4	0.16	0.52	4.00	0.010	0.010	0.510	0.11	0.20	<0.003	0.037	0.0030
lab-5	0.17	0.50	3.96	0.010	0.009	0.027	0.11	0.02	<0.003	0.032	<0.0005
ref	0.44	0.30	0.80	0.015	0.022	—	1.15	0.19	—	—	—

* C, S determined with ‘Leco’ combustion analyses.

**Table 2:** Overview of the pγ-GS of the investigated alloys and heat treatments.

*T*_d_	lab-1	lab-2	lab-3	lab-4	lab-5
[${}^{\circ }C$]	[μm]	[μm]	[μm]	[μm]	[μm]
1200	56	39	58	65	75
1100	47	45	52	38	51
1000	38	33	62	43	52
900	36	40	81	130	77
800	31	39	88	139	79

**Table 3:** Maximum equivalent stresses σ_e_ for deformations from 800 ${}^{\circ }C$–1200 ${}^{\circ }C$.

*T*_d_	lab-1	lab-2	lab-3	lab-4	lab-5	ref
[${}^{\circ }C$]	[MPa]	[MPa]	[MPa]	[MPa]	[MPa]	[MPa]
1200	65.2	64.8	69.0	69.0	70.0	68.8
1100	80.5	93.4	98.1	103.7	88.3	87.8
1000	134.3	128.7	144.6	137.8	121.3	129.7
900	193.0	185.5	192.7	193.8	173.8	194.5
800	246.2	246.7	238.8	259.6	229.4	—

**Table 4:** Measured Vickers hardness values for the investigated alloys and heat treatments. The mean square error is given as well.

*T*_d_	lab-1	lab-2	lab-3	lab-4	lab-5
[${}^{\circ }C$]	[HV10]	[HV10]	[HV10]	[HV10]	[HV10]
1200	457 ± 10	431 ± 6	447 ± 9	433 ± 3	434 ± 4
1100	462 ± 11	438 ± 6	443 ± 6	422 ± 7	452 ± 3
1000	467 ± 5	432 ± 6	455 ± 7	444 ± 1	450 ± 3
900	482 ± 10	455 ± 1	463 ± 6	448 ± 5	471 ± 5
800	490 ± 5	463 ± 2	477 ± 4	471 ± 5	486 ± 9

## Abbreviations

The following abbreviations are used in this manuscript:

Q + T	Quenched and Tempered Steel
PHFP	Precipitation Hardened Ferritic Perlitic Steel
HDB	High ductile Bainite
TRIP	Transformation induced plasticity
AHD	Air-hardening ductile
MMnS	Medium Manganese Steels
DRX	Dynamic Recrystallization
DRV	Dynamic Recovery
CCT	Continuous Cooling Transformation
